# Arthropod Containment Guidelines, Version 3.2

**DOI:** 10.1089/vbz.2018.2431

**Published:** 2019-02-26

**Authors:** American Committee of Medical Entomology; American Society of Tropical Medicine and Hygiene

**Keywords:** guidelines, vector-borne disease, laboratory safety, risk assessment, containment, arthropod

## Abstract

The Arthropod Containment Guidelines are a product of the work of the American Committee of Medical Entomology, a subcommittee of the American Society of Tropical Medicine and Hygiene. The guidelines provide a reference for research laboratories to assess risk and establish protocols for the safe handling of arthropod vectors of human and animal disease agents. The guidelines were originally published in 2004 and have been updated here to reflect the spectrum of vector taxa under investigation, and the demands of working with vector arthropods in the context of the Select Agent Rule.

## Introduction

Laboratories in which living arthropods are reared and maintained for research purposes have been in existence for decades with few reports of harm to their workers or to the communities in which they are located. Many of these organisms are associated with potential risks should they escape, since many are vectors of infectious human diseases. When they are experimentally infected with a human pathogen, the arthropods represent an immediate risk to those who come into contact with them. Even when they are uninfected, they can represent a risk to the community if, by escaping, they become the crucial link completing the transmission cycle for a pathogen.

There are two prominent examples of initially small exotic vector introductions that resulted in significant disease increases. In the early 1900s, anopheline mosquitoes were drastically reduced in northeastern South America because of eradication campaigns. The concomitant drop in incidence of malaria and other human infectious diseases was reversed after *Anopheles gambiae* was discovered in the port city of Natal, Brazil, in 1930 (Soper and Wilson 1943). The African malaria vector was accidentally introduced into the area, probably by rapid marine mail service. Although the release was not from a laboratory, the introduction of a highly efficient vector is widely thought to be responsible for the resurgence of malaria in Brazil. Fortunately, an aggressive effort to eradicate *An. gambiae* by conventional means was successful.

A second example concerns the current distribution of the Chagas disease vector, *Rhodnius prolixus*, throughout rural Central America. Whereas this insect is considered indigenous to northern South America, it is thought to have been introduced into Central America through a laboratory escape that occurred in El Salvador in 1915 (Schofield 2000). While there are a number of other triatomine species throughout Central America that transmit the agent of Chagas disease to humans, the establishment of *R. prolixus* was especially important because of its close association with humans and domestic dwellings. *R. prolixus* is considered the most important vector of Chagas disease in Central America and the species targeted for elimination by the Central American Chagas Disease Control Initiative.

Past editions of the reference manual “Biosafety in Microbiological and Biomedical Laboratories” (BMBL) (Centers for Disease Control and Prevention [CDC] 1999) noted that despite the long history of accidental laboratory infections of workers and their immediate contacts, “laboratories working with infectious agents have not been shown to represent a threat to the community” referring to reviews of laboratory-acquired infections at CDC and the National Animal Disease Center which documented that there were no instances of secondary transmission (Richardson 1973, Sullivan et al. 1978). Nevertheless, the historical accidental introductions *An. gambiae* and *R. prolixus* suggest the possibility for community impacts of introduced arthropod vectors, even when uninfected.

The advent of transgenic technology in which vector arthropods are now routinely genetically modified requires arthropod containment recommendations with a particular view to preventing inadvertent escape and establishment. These guidelines are intended to do that. They have been drafted by a subcommittee of the American Committee of Medical Entomology (ACME) and circulated widely among medical entomology professionals. The Committee membership and drafting procedures are summarized in Appendix 1–3. These guidelines represent the position of the committee as a whole, not that of all individuals nor of the membership's individual institutions.

Transgenic arthropods and those containing microbes modified by recombinant DNA technology are addressed here solely in the context of public health significance. The emphasis is therefore on the phenotypic changes resulting from the modification rather than ecological and environmental issues, which are addressed elsewhere (WHO/TDR and FNIH 2014, Marshall 2010).

## Background

### Several documents particularly influenced the effort to create these guidelines and shaped their content

#### 1980

The Subcommittee on Arboviral Laboratory Safety, a subcommittee of the American Committee on Arthropod-Borne Viruses (ACAV), a sister organization of ACME, published guidelines in the *American Journal of Tropical Medicine and Hygiene* (The Subcommittee on Arbovirus Laboratory Safety 1980). These were developed on the basis of a survey of almost 600 laboratories and covered a wide range of pathogenic viruses. They addressed issues relevant to the safety of laboratory workers and assigned each virus to one of four “levels of containment and practice” based on the severity of the associated human disease, the method of transmission, and the amount of experience handling the organism. The document addresses containment of the arthropod vector for each level, but only when it is infected by the viral agent. It did not address nonviral (*e.g*., eukaryotic or bacterial) agents or their vectors, transgenic animals, nor did it take into account biological containment that may be provided by the climate or other characteristics of the location in which the research is conducted.

#### 1984

BMBL was published in response to a series of surveys, beginning, for example, with reports of laboratory-acquired infections (Sulkin et al. 1949, Richardson 1973, Sullivan et al. 1978). Now in its fifth edition, this Public Health Service document describes the practices, facilities, and equipment suggested to safely work with potentially dangerous agents in a laboratory (Centers for Disease Control and Prevention [CDC] 2009).

#### 1995

The American Mosquito Control Association adopted a position that containment of genetically manipulated arthropods be addressed by funding and regulatory agencies. A letter sent to the Directors of the National Science Foundation, the CDC, the National Institute of Allergy and Infectious Diseases, the Pan American Health Organization, and the Administrator of the U.S. Department of Agriculture (USDA) urged that “guidelines for research be developed to ensure that no exotic agents are accidentally released, and to ascertain the potential for the released organisms to alter vector-borne disease transmission patterns.”

#### 1996

Hunt and Tabachnick (1996) prepared a forum article on containment of very small arthropod vectors. This provided a means to test the efficacy of containment and addressed the peculiar containment needs of laboratories working with arthropods too small to be restricted by conventional insectary precautions.

Higgs and Beaty (1996) authored a chapter entitled “Rearing and Containment of Mosquito Vectors.” The chapter gives practical advice and illustrations for the design, construction, and operation of insectaries.

#### 1997

The Molecular Biology of Disease Vectors was published (Crampton et al. 1997). It contains several chapters describing methods for the experimental infection of a wide variety of insects. Although most of the emphasis of this textbook is methodological, safety aspects are also addressed.

#### 1998

The USDA, through its Animal and Plant Health Inspection Service (APHIS), developed draft guidelines for the containment of nonindigenous, phytophagous arthropods and their parasitoids. These guidelines include standards for construction, equipment, and operations when handling arthropod pests of plants. While the document focuses on preventing environmental detriment, the general containment principles for this class of arthropods are relevant to hematophagous arthropods.

#### 1999

The Department of Health and Human Services, through the National Institutes of Health (NIH), issued revised guidelines for the safe handling of organisms that contain recombinant DNA, including arthropods (NIH Guidelines 2016). Paralleling the BMBL, these guidelines also specify that an institutional biosafety committee (IBC) review all nonexempt research protocols involving recombinant DNA and approve the level and implementation of appropriate containment. Section III.D.4 of the Guidelines specifically addresses arthropods that contain recombinant DNA and assign them to a minimum of biosafety level (BSL)-2.

The American Society of Tropical Medicine and Hygiene (ASTMH), ACME, adopted a resolution to develop these Arthropod Containment Guidelines. The guideline's form is directly based on the structure and wording of BMBL (American Committee of Medical Entomology and American Society of Tropical Medicine and Hygiene 2003) (see Appendix 2).

## Intent

This document supplements BMBL by providing safety principles for handling arthropod vectors. Readers should refer to BMBL for standard and special microbiological practices appropriate for the agents with which they work. These have been repeated here when they would apply to the vector alone as well as the agents. Throughout, the authors have attempted to formulate guidelines that are consistent with those of BMBL, yet recognize the fact that biological containment (*i.e*., location- and season-specific fate of escaped arthropods in the environment) significantly confounds risk assessment and therefore appropriate safety practices. Furthermore, the flying, crawling, burrowing, and reclusive habits of arthropods, combined with the agents they may carry, introduce an element of risk-increasing behavior not covered by BMBL.

This document describes the arthropod handling practices, safety equipment, and facilities constituting Arthropod Containment Levels 1–4 (ACL-1 to -4). These are recommended by the ASTMH/ACME for work with a variety of uninfected arthropods and those carrying infectious agents, and for work with transgenic vector arthropods in laboratory settings. The principles of risk assessment, specific practices, and equipment will also be useful in nontraditional arthropod research settings such as tents, greenhouses, and outdoor cages. Field sites at which research with such arthropods is conducted are defined by the type and duration of activities that occur there and the risks to the participants and inhabitants, but they are not the focus of the document. Although plant pathologists and entomologists may find this document useful, the focus of this document is on arthropods that transmit pathogens of public health importance. More details specifying the arthropods that are generally excluded from these guidelines can be found under “Arthropod Containment Levels.”

This document is strictly concerned with laboratory research that involves arthropods of public health importance and risks associated with pathogen transmission. Arthropods to be considered include among others: insects (Diptera—mosquitoes, tsetse flies, black flies, sand flies, midges; Hemiptera—kissing bugs; Phthiraptera—lice; Siphonaptera—fleas) and arachnids (Acari—ticks, mites). All life cycle stages, eggs, larvae, nymphs, adults, must be considered under the term arthropod. The small size, highly motile characteristics of some arthropods (especially flying and jumping), and relative long life and resistance of some stages make the containment of arthropods a unique problem. The diversity of these organisms and their complex life cycles often mean that procedures and practices to safely contain the animal are species-specific. Conversely, the specific culture requirements of some species make maintenance difficult, but containment relatively straightforward since they cannot survive outside of the preferred habitat. Although noninsects (ticks and mites) are also considered here, the designated area in which these organisms are maintained and cultured will hereafter be referred to as an insectary.

From the above, it follows that many arthropods (*e.g*., fruit flies, cockroaches, and various Lepidoptera and Coleoptera) that have been collected locally, or have been purchased from pet stores or commercial vendors for study or educational purposes, are usually exempt from these guidelines. However, experiments planned with nonvector arthropods that are deliberately infected with a disease agent might be informed by these guidelines.

Arthropods are an important educational tool in many schools and colleges, and are collected and cultured for many different purposes. These guidelines should not impact interest in and research on uninfected arthropods that pose no danger to the surrounding environment and public health. In these circumstances, maintenance and rearing techniques are at the discretion of the student or instructor, and do not fall within the containment criteria described in these guidelines.

It should be noted that although these guidelines are intended mainly for U.S. research institutions and those programs in other countries receiving NIH funds, there are many institutions outside of the United States or the European Union, particularly in resource-limited sites, where administrative constructs such as institutional animal care and use committee (IACUC), IBC, biosafety officers, and occupational health clinics do not exist. Small institutions even in the United States may not have the infrastructure or funds to renovate laboratories, and therefore, any alternatives to mitigate these realities have to be thoroughly vetted in a risk assessment to ensure that research on vector arthropods is safe and fully contained.

These recommendations are advisory. They provide a voluntary guide or code of practice as well as principles for upgrading operations. They also offer a guide and reference in the construction of new laboratory facilities or the renovation of existing facilities. The application of these recommendations to any particular laboratory must be based on a risk assessment of the particular vector species, disease agents, activities, and geographic location of the laboratory.

## Field Sites

Sites at which vector arthropod research is conducted necessarily span a wide range of sophistication and infrastructure from modern structures that are clearly “laboratories”—the primary focus of this document—to primitive field sites that do not. To clearly define the characteristics that distinguish a laboratory from a field site, we describe characteristics of these activities to enable researchers, IBCs, and granting organizations to determine whether these laboratory guidelines apply at all. A field site is a temporary facility, the work is performed with indigenous arthropod vectors that are collected locally, and in the event of escape, there is likely only a small risk that released arthropods would modify the genetic structure of resident arthropod populations or increase the risk of human or animal infection with locally transmitted pathogens. Maintenance of indigenous vectors for local research may occur in these facilities. Field laboratories usually do not require structural modification of existing buildings for vector containment, rather arthropod escape is minimized by appropriate primary containment (caging) and handling practices.

Field laboratories are often located in places where the species under study is involved in pathogen transmission. Because vectors brought from the field into the laboratory may be naturally infected with a pathogen, appropriate precautions should be taken to minimize researcher exposure to infectious organisms. On the contrary, the risk within the laboratory by manipulating collected samples may be less than working outside. The risk assessment process will determine what precautions, if any, are warranted, for example, vaccines, prophylaxis, and repellents.

## Authorities

Importation and transport of exotic arthropods of public health importance fall under the purview of the Public Health Service/CDC, Office of Health and Safety (United States Public Health Service [USPHS] 42 Code of Federal Regulations [CFR], Part 71.54). Many arthropods transmit both human and animal diseases and may therefore also be regulated by the USDA APHIS. Arthropods modified by recombinant DNA methods or containing similarly modified microbes are addressed in the NIH Guidelines and may be regulated by the U.S. Environmental Protection Agency (EPA) and/or the U.S. Food and Drug Administration (FDA).

## Risk Assessment for Arthropod Vectors

The intent of this section is to provide guidance and to establish a framework for selecting the appropriate ACL (facilities, equipment, and practices), reducing risks of release and exposure of laboratory workers and the public to a vector and associated agents.

“Risk” in the context of this document implies the probability that harm, injury, or disease will occur among laboratorians or the general public because of accidental release of a competent disease vector and/or associated agents. In the context of vector research laboratories, risk assessment considers two kinds of effects: direct effects such as biting, infestations, and myiasis, and indirect effects of morbidity and mortality due to the pathogens transmitted. The latter is by far of higher concern, and direct effects are not considered here. Therefore, in this document, ACLs are directly correlated with the appropriate BSL of the agents with which they are naturally or experimentally infected or may transmit in the event of accidental release (see BMBL Section VI).

While the focus of this document is human health risk, effects on animals because of arthropods known to transmit animal disease are to be considered. Researchers are encouraged to consult with the U.S. Fish and Wildlife Service and USDA-APHIS regarding risks and regulation before completing a risk assessment.

The laboratory director or principal investigator (PI) has primary responsibility for assessing risks to set the appropriate BSL for the work. This is done in close collaboration with the IBC, if there is one, to ensure compliance with established guidelines and regulations. Development and review of the risk assessment and the planned safety precautions by consultation with experts in the biology and public health significance of the arthropod are essential.

In performing a qualitative risk assessment, all the risk factors are first identified and explored considering related information available such as BMBL, the NIH Guidelines, the Canadian Laboratory Biosafety Guidelines (Public Health Agency of Canada 2004), the WHO Biosafety Guidelines (World Health Organization (WHO) 2004), and the ACAV Catalogue of Arboviruses (Centers for Disease Control and Prevention [CDC] 1985). In many cases, one must rely on other sources of information such as field data, the literature concerning aspects of vector competence, and environmental requirements through consultation with recognized experts in arthropod and pathogen relationships.

The greatest challenge of risk assessment lies in those cases where complete information on these factors is unavailable. A conservative approach is advisable when insufficient information forces subjective judgment.

## Principles of Risk Assessment

Arthropod risk assessment is primarily a qualitative judgment that cannot be based on a prescribed algorithm. Several factors must be considered in combination: the agents transmitted, whether the arthropod is or may be infected, the mobility and longevity of the arthropod, its reproductive potential, biological containment, and epidemiological factors influencing transmission in the proposed location or region at risk. Arthropod vectors of infectious agents can be assigned to the following discrete categories. Each category has a range of risks that need to be assessed.

### Arthropods known to be free of specific pathogens

Risk from these materials to laboratorians is similar to that experienced by the general public: nuisance due to consequences of escape and temporary or permanent establishment. Consequently, the public health risk is likely to be low unless epidemiological conditions exist that could reasonably be expected to result in an increase in transmission of an endemic disease in that particular region, or establishment of the released vector leads to significant risk of future transmission potential for an exotic pathogen. In the event that establishment is likely, the arthropod must be handled under more stringent containment conditions.

If an accidental release occurs, followed by even transient establishment of an uninfected arthropod, the probability of increased transmission must be considered in the context of the location in which the work will be performed or in regions to which escaped arthropods could likely migrate. For example, escape of an exotic malaria vector in a malarious region has a significantly higher probability of increasing transmission and therefore a higher risk than escape in a nonmalarious region. The pathogenicity of the agent and availability of treatments and drugs should also be considered.

Answers to the following questions will affect the level of risk due to accidental escape of uninfected arthropods:

- Is the arthropod species already established in the locale?
- If the arthropod is exotic, is it likely that the arthropod would become temporarily or permanently established in the event of accidental escape?
- Does the arthropod have a known or characterized insecticide-resistant genotype or phenotype?
- Could the arthropod be realistically controlled or locally eradicated by traditional methods (*e.g*., spraying, trapping) in the event of escape?
- Are the agents that the arthropod is known to transmit cycling in the locale, or has the agent been present in the past?
- Are agents that the arthropod could reasonably be expected to transmit to animals present in the locale?
- Would accidental release of the arthropod significantly increase the risk to humans and animals above that already in existence in the event of introduction of exotic pathogens in the area?
- In the case of zoonotic diseases, does the animal reservoir exist in the locale, and, if so, what is the infection status?
- Was the exotic arthropod derived from a subpopulation (strain, geographically distinct form) whose phenotype is known or suspected to vary in ways that could reasonably be expected to significantly increase its vector competence? If so, it should be handled under the more stringent conditions within ACL-2 (described below) even if uninfected.
- Are disabled strains available, whose viability after escape would be limited (*e.g*., eye-color mutants, cold-sensitive)?

### Arthropods known to contain specific pathogens

Arthropods that are known to be, or reasonably suspected of being, infected with infectious agents always have risks that must be identified, and appropriate precautions must be taken for worker and public health safety. The characteristics of most known infectious agents have been well-defined and are the starting point for determining risk from these arthropods. Information useful to risk assessment can be obtained from laboratory investigations, disease surveillance, and epidemiological studies. Infectious agents known to have caused laboratory-associated infections are included in the BMBL agent summary statements (Section VII). Other sources include the American Public Health Association's manual, Control of Communicable Diseases (Benenson 1995). Literature reviews on laboratory-acquired infections also may be helpful (Sulkin et al. 1949, Richardson 1973, Sullivan et al. 1978).

The pathogenicity of the infectious or suspected infectious agent, including disease incidence and severity (*i.e*., mild morbidity versus high mortality, acute versus chronic disease), is the most important consideration in assessing the risk due to accidental exposure to an infected arthropod vector. As the initial criterion, it is clear that the more severe the potentially acquired disease, the higher the risk.

Readers will observe that ACL-2 has broad latitude in specific practices. This reflects, in part, the widely differing degrees of effects of arthropod-borne agents, many of which fall within the BSL-2 level. Considerable variation in morbidity and mortality exists within the level 2 classification. For example, level 2 arboviruses are exemplified by La Crosse virus with a 1% or less mortality rate and limited, mild neurological sequelae. Higher containment levels are recommended for agents that cause disease in humans considered potentially severe, life threatening, or cause residual damage. Our general approach in formulating these guidelines has been to include a wide range of ACL-2 features that reflect this broad range of agent potency. Moreover, the possible natural and artificial modes of infection (*e.g*., parenteral, airborne, ingestion) of the agent are considered. This is essential to prevent infections in laboratorians.

The established availability of an effective prophylaxis or therapeutic intervention is another essential factor to be considered. The most common form of prophylaxis is immunization with an effective vaccine. In some instances, immunization may affect the BSL or ACL; for example, Central European Tick Borne Encephalitis virus has been reclassified to BSL-3/ABLS3 (from BSL-4/animal biosafety level [ABS]-4) when laboratory personnel are vaccinated (BMBL5, p. 236). Although IBCs once could permit certain tasks and procedures with eastern equine encephalitis virus (EEEV) to be conducted at BSL-2 (from BSL-3) when personnel were vaccinated, recent regulations appear to prohibit such a downgrading (www.selectagents.gov/guidance-encephalitis.html). At any rate, the availability of therapeutics and vaccines only serves as an additional layer of protection beyond engineering controls, proper practices, and procedures, and the use of personal protective equipment. Occasionally, immunization or therapeutic intervention (antibiotic or antiviral therapy) may be particularly important in field conditions. The offer of immunizations is part of risk management to protect laboratory workers and vaccination may be demanded, as a condition of employment, for any laboratory worker working with yellow fever virus, or any pathogens for which an efficacious vaccine is available. Such a policy may vary from institution to institution.

Medical surveillance is encouraged to ensure that the instituted safeguards provide the expected health outcomes. Surveillance may include serum banking, monitoring employee health status, and participating in postexposure management. However, as with vaccination, the implementation of medical surveillance may vary by institution. In the arthropod vector laboratory, this must be combined with regular monitoring for escaped arthropods, for example, inventory of infected arthropods, an effective arthropod trapping program, and regular inspection of the facilities for disrepair that could result in escape.

Risk assessment must also include an evaluation of the experience and skill level of at-risk personnel such as laboratorians, maintenance, housekeeping, and animal care personnel. Additional education may be necessary to ensure the safety of persons working at each BSL.

### Arthropods containing unknown infectious agents or whose status is uncertain: diagnostic samples

The challenge here is to establish the most appropriate containment level with the limited information available. Some questions that may help in this risk assessment include the following:

- Why is an infectious agent suspected?
- What route of transmission is indicated?
- Are agents that the arthropod transmits transferred horizontally?
- Are there reasons to believe that a novel or unknown agent is present?
- What epidemiologic data are available?
- What is the morbidity or mortality rate associated with the agent?

Bringing field-collected arthropods into a laboratory may be associated with the possibility that personnel who would not otherwise be exposed to any risk because they do not work in field sites might be placed at risk. Researchers working in field sites often handle arthropods of unknown infection status under conditions that do not allow implementation of typical laboratory precautions, but they are in the field and understand that their actions may expose them to associated risks. Answers to the questions above will assist researchers in determining potential risks and reasonable solutions.

Although the most conservative approach would be to consider all field-collected arthropods as potentially infectious, it should be recognized that the prevalence of infection for most vector/pathogen relationships is small. With many arboviruses, a prevalence of 1 in 1000 may be typical. Infection may not be viable, or the quantum of infection (potential dose) may be below that required to produce productive infection. There may be strains or populations of infectious agents that are avirulent. Delivery of an agent by contamination (exposure to aerosol or arthropod tissues) may be significantly less efficient in producing infection than by bite, in which immunosuppressive and anti-inflammatory molecules in salivary secretions may promote infection. For those agents with complex arthropod developmental cycles, the stages that are present may not be capable of productive infection. There are thus many factors involved in assessing risk associated with unknown or diagnostic samples, and a conservative approach may not be warranted.

The recent guidelines for clinical laboratories (Centers for Disease Control and Prevention [CDC] 2012) provide an excellent approach to risk assessment that may be applied to diagnostic or unknown arthropod samples. Diagnostic clinical samples (humans or animals) are mainly handled at BSL-2, although specific scenarios (suspected viral hemorrhagic fever, tuberculosis, generation of aerosols, larger volumes) may require additional safety practices and procedures. This publication provides particularly detailed and nuanced discussion of risk assessment in the clinical diagnostic setting that places into context a risk assessment that allows for other than the very conservative recommendation that if an arthropod is suspected to contain a certain agent, the corresponding BSL should be applied.

### Vector arthropods containing recombinant DNA molecules

The purpose of this section is to present principles of risk assessment of vector arthropods that have been genetically modified, typically via recombinant DNA technology. This includes both vector arthropods that contain modified microbes and which themselves are genetically modified. These principles primarily address the public health significance of the modified organisms rather than environmental concerns. These technologies continue to evolve rapidly, and experimental procedures designed to derive novel modified symbionts and recombinant arthropods are becoming commonplace.

The NIH Guidelines (2016) are a key reference in establishing an appropriate BSL for work involving recombinant organisms, including microorganisms for use in arthropods and genetically modified arthropods themselves. In selecting an appropriate ACL for such work, the greatest challenge is to evaluate the potential biohazard change resulting from a particular genetic modification relative to the unmodified arthropod. In the context of public health, the selection of an appropriate level begins by establishing the phenotypic change in the arthropod and/or microorganism due to DNA manipulation, and potential impact of escaped arthropods containing the modification.

Among the points to consider in work with recombinant arthropod vectors and those containing recombinant microbes are the following:

- Does the inserted gene encode a product known or likely to alter the vector capacity or competence for pathogens it is known to transmit?
- Does the inserted gene cause phenotypic changes that could significantly affect the ability to control the arthropod if there were an accidental escape, for example, an insecticide resistance marker?
- Does the modification have the potential to alter the range or seasonal abundance of the arthropod?
- If so, would the new range increase the likelihood that the vector could transmit new pathogens?
- Is the modified strain disabled in a way that viability after escape would be limited (*e.g*., eye-color mutants, cold-sensitive)?
- Does the modification have the potential to increase the reproductive capacity of the arthropod that carries it?
- Is the phenotype conferred by the modification, including its marker and other expressed genes, if any, consistently expressed after numerous generations of propagation?
- Is the modification undergoing rearrangement or other mutations at a measurable rate?
- Can the DNA transgene vector be mobilized in natural populations?
- Is the host range of the symbiont known?
- Would the modified symbiont pose increased risk to immunocompromised persons relative to the native symbiont?
- Is the entire sequence of the DNA insertion known, and are the coding sequences defined?
- Is horizontal transfer of the transgene to other microbes with which the modified microbe is likely to come into contact possible?
- Is the original insertion site known so that stability can be assessed later?

This list of questions is not meant to be exhaustive; expert discussions and guidelines are now available for this particular aspect of vector biology (Booij 2013, Benedict 2014, WHO/TDR and FNIH 2014). Rather, the list illustrates the information needed to provide an accurate and conservative assessment of risk to judge the appropriate containment level. Since in many cases the answers to the above questions will not be definitive, it is important that the organization has a properly constituted and informed IBC, as outlined in the NIH Guidelines, to evaluate risk assessment and provide prudent adherence to appropriate safety guidelines for the assigned risk.

## Arthropod Containment Levels

The Arthropod Containment Level (ACL) guidelines were first published over a decade ago (American Committee of Medical Entomology and American Society of Tropical Medicine and Hygiene 2003) (see Appendix 2). In the interim, new pathogens have been described, BSL-3 laboratories have proliferated, and the Select Agent Rule has evolved to include thorough biosafety assessment in addition to biosecurity. This revision of the guidelines was initiated with the objective of clarifying and modifying or strengthening recommendations to ensure the safety of researchers as well as the community without hindering the pursuit of knowledge and development of vector control methods and life-saving solutions to vector-borne disease issues. We particularly emphasize the absolute need for local risk assessments to complement the regulatory framework as opposed to a checklist approach based on these guidelines. Where applicable, we point out critical aspects of the Select Agent Rule.

When arthropods are used in research, facilities, trained staff, and established practices must be in place to ensure appropriate safety, and the protection of health and well-being of workers and the environment. This article provides guidelines for laboratory work with hematophagous arthropods and arthropod vectors of pathogenic agents, and was originally drafted in response to concerns related to the consequences of an accidental release of arthropods. These consequences are basically answering the question “What happens if the arthropod escapes?” and the suggested containment levels address the question “How do we prevent escape?” When working with a vector in a particular set of circumstances (Table 1), a certain containment level may be recommended. It should be noted, though, that the local IBC is an essential component in establishing the appropriate ACL. It is responsible for reviewing a research protocol and decides at what level of containment the experiments must be performed. Accordingly, a “one-size-fits-all” checklist based on these guidelines is not appropriate for risk assessment; local IBCs may, for example, allow research to proceed even if it is not possible to adhere to all recommended guidelines, such as those of the BMBL. In such cases, alternative means of mitigating risk would be evaluated in the site-specific risk assessment (Cauchetaux and Mathot 2005).

**Table 1.** Summary of Arthropod Containment Levels

*Anthropod containment level (ACL)*	*1*		*2*	*3*^a^	*4*^a^
*Arthropods free of specific pathogens*	*Indigenous/no change in local fauna*	*Exotic/inviable or transient only*	*Exotic with establishment potential or transgenic*	*n/a*	*n/a*
Infection status	Up to BSL-1		Up to BSL-2	Up to BSL-3	Up to BSL-4
Practices	ACL-1 standard handling practices		ACL-2 and BSL-2 limited access, training, signage, containment, and disposal	ACL-3 and BSL-3 restricted access, training, appropriate PPE, signage, containment, disposal, record-keeping^a^	ACL-4 with BSL-4 isolation, training, appropriate PPE, signage, containment, disposal, record-keeping^a^
Primary barriers	Species-appropriate containers		Appropriate PPE, escape-proof containers	Appropriate PPE, escape-proof containers, pesticide available for emergency use^a^	Appropriate PPE, escape-proof containers, pesticide available for emergency use^a^
Secondary barriers			BSL-2 facilities, breeding sites, and harborage minimized, pest control	BSL-3 facilities, biological safety cabinets, other physical containment devices, pest control^a^	BSL-4 and facility-specific procedures and equipment for arthropod handling while wearing positive pressure containment suit^a^

General guidelines for best laboratory containment practices are shown for vector species of arthropod that are uninfected (a*bove the bold line*) or infected (*below the bold line*) according to biosafety and ACLs. Indigenous species are those species whose current range includes the research location. All others are considered exotic. For uninfected arthropods, containment guidelines take into account the consequences of accidental escape from a laboratory, in which the arthropod would be (1) inviable as a result of exposure to unfavorable conditions; (2) transient because conditions vary such that the arthropod would die during typical year climate cycle; or (3) has potential for establishment because escaped arthropods could reasonably be expected to persist through a typical climatic year. Arthropod containment specifics for each BSL should always be reviewed in the context of a laboratory-, vector-, and pathogen-specific risk assessment that is based on consultation between the investigator and the appropriate institutional oversight committee(s) and according to the constraints of the infrastructure available.

^a^ Additional restrictions apply for work with arthropods in association with Select Agents.

ACL, arthropod containment level; BSL, biosafety level; PPE, personal protective equipment.

One significant practice emphasized as a result of the original ACL guidelines is that when an arthropod is infected with an agent, the containment level required is automatically increased to at least that required for the agent, regardless of factors such as the competence of that arthropod for the particular pathogen. Containment levels for most infectious agents, the rationale for categorizing them at such containment levels, and recommendations for their safe manipulation are specified in the BMBL. An example is the use of male mosquitoes to propagate dengue viruses. Although they cannot transmit by bite, the presence of the viable agent requires that they be held at BSL-2. Injection of male mosquitoes with dengue virus or homogenizing them afterward to assay for viral replication might expose personnel to infection. Even if the actual infection status of each individual male mosquito inoculated with dengue virus is not known, the standard of practice with diagnostic materials that may contain an infectious agent is to manipulate them at BSL-2.

Another practical example of the utility of the ACL guidelines relates to the use of exotic arthropods. Biological introductions are to be prevented by all reasonable means, and handling exotic arthropods at ACL-2 or higher greatly reduces that risk. At any rate, even before the formal ACL guidelines, USDA import and possession permits would stipulate the equivalent of the ACL-2 recommendations for use of most exotic arthropods. It is impossible to prescribe universal levels of containment for a particular species since the risks associated with accidental release from a laboratory are determined by several factors, for example, the climate at the facility and history of transmission in that location. For example, the accidental release of an uninfected anthropophilic tropical vector species, during the winter in Wisconsin, should be considered significantly less of a risk than the release of the same species in a tropical area in which it could become permanently established and act as a bridge vector of an established zoonotic pathogen to humans. Thus, one of a number of possible approaches to reduce the risk of a release might be to perform relatively high-risk experiments during the winter when any escaped arthropods would quickly be killed by adverse environmental conditions; or a rainforest species could be used in a laboratory located in a xeric environment. IBCs might even use such biological considerations to “downgrade” a particular protocol from ACL-3 to ACL-2, providing that experiments are performed during a particular period or in a particular site. On the contrary, the possibility of zoonotic transmission or promoting a risk to domestic animal production means that we have to consider in risk analysis those pathogens that are predominantly an animal health issue; USDA Guidelines may need to be considered when assigning a containment level to a particular vector species.

The current research and applied focus on genetically modified arthropods also mandate recommendations that reduce the risk of accidental environmental release. Although the ACL guidelines can be used as one basis for risk assessment and mitigation, the science of transgenic vectors is an evolving one and the reader is directed to specific documents that provide experience-based guidance on such experiments (Centers for Disease Control and Prevention [CDC] 1985, Benenson 1995).

Although specific recommendations are not made herein because they depend on site-specific details, all IBC protocols should outline an emergency response procedure that is appropriate in case of an accidental release. The ideal response would be one in which all released arthropods are killed almost immediately after the escape. This may be impossible if the escaped arthropods get outside of the laboratory, and hence, redundant facility and practice barriers are recommended to maximize the opportunities for location and destruction of the escapees.

## Arthropod Containment Level 1

ACL-1 is suitable for work with uninfected arthropod vectors or those infected with a nonpathogen, including (1) arthropods that are already present in the local geographic region regardless of whether there is active vector-borne disease transmission in the locale and (2) exotic arthropods that on escape would be nonviable or become only temporarily established in areas not having active vector-borne disease transmission. This category would include most educational use of arthropod vectors. A summary of the containment levels is provided in Table 1.

### Standard practices

#### Location of arthropods

Furniture and incubators containing arthropods are located in such a way that accidental contact and release are minimized. This may be achieved by locating arthropods out of the flow of general traffic, avoiding hallways, or placing them in closets.

#### Supply storage

The area is maintained to allow detection of escaped arthropods. For example, materials unrelated to arthropod rearing and experimentation (*e.g*., plants, unused containers, clutter) that provide breeding sites and harborages are minimized.

#### General arthropod elimination

Accidental sources of arthropods from within the insectary are eliminated. This may be accomplished by cleaning work surfaces after a spill of materials, including soil or water that might contain viable eggs. For example, personnel in mosquito laboratories should immediately eliminate any standing water.

#### Primary container cleaning and disinfestation

Practices should be in place such that arthropods do not escape by inadvertent disposal in primary containers. Cages and other culture containers are appropriately cleaned to prevent arthropod survival and escape (*e.g*., heated to, or chilled below, lethal temperature).

#### Primary container construction

Cages used to hold arthropods effectively prevent escape of all stages. Screened mesh, if used, is durable and of a size appropriate to prevent escape. Nonbreakable cages are recommended. Bags, rearing trays, and so on effectively prevent leakage and escape.

#### Disposal of arthropods

All life stages of arthropods must be killed before disposal. Arthropods may be killed with hot water or freezing before flushing down drains or placed into trash bags.

#### Primary container identification and labeling

Arthropods are identified with descriptive labels to include the species, strain/origin, date of collection, responsible investigator, and so on; labels are firmly attached to the container (and cover if removable). Vessels containing stages with limited mobility (*e.g*., eggs, pupae, hibernating adults) are likewise labeled and (if applicable) housed or stored to prevent progression to, and escape of, a mobile life stage.

#### Prevention of accidental dispersal on persons or via sewer

Personnel take appropriate precautions to prevent transport or dissemination of live mobile arthropods from the insectary by practicing appropriate disposal methods and preventing escapees at every level of containment (primary container, environmental chamber, laboratory, etc) to prevent dispersal on persons.

#### Escaped arthropod monitoring

Investigators assess whether escapes are occurring. An effective arthropod trapping program is recommended to monitor the escape prevention program.

#### Pest exclusion program

A program to prevent the entrance of wild arthropods (*e.g*., houseflies, cockroaches, spiders) and rodents effectively precludes predation, contamination, and possible inadvertent infection.

#### Source and harborage reduction

Harborage and breeding areas are reduced as appropriate. Furniture and racks are minimized and can be easily moved to permit cleaning and location of escaped arthropods.

#### Notification and signage

Persons entering the area may be made aware of the presence of arthropod vector species by signage if recommended by an institutional research oversight committee.

### Special practices: vertebrate animal use

#### Institutional approval

Investigators should consult with their institutional research oversight office if vertebrate animals will be used to feed hematophagous arthropods. The requirement for IACUC and/or IBC review is an institutional decision, although highly recommended.

#### Housing of vertebrate animals

Animals used as hosts or blood sources should be housed according to institutional laboratory animal guidelines. If necessary, vertebrate animals may be housed within the insectary but need to be adequately protected from access by escaped arthropods. Animals not necessary for maintaining arthropods should not be accessible to hematophagous arthropods in the laboratory setting.

#### Containment during blood feeding

Special considerations should be taken when hematophagous arthropods are fed on host animals. The primary container must be sufficiently robust to prevent escape during feeding. When handling/removing vertebrate animals after exposure to arthropods, precautions must be taken to prevent arthropod escape through screens, covers, and by flying. Host animals are inspected closely (*e.g*., concealment in fur, ears, axillae, or other possible hiding places). Finally, all precautions should be taken to prevent arthropods fed on host animals from accidental transfer to host cages and therefore dispersal outside of containment, if animals and their cages are returned to a holding room.

#### Blood source

The blood source should be considered a possible source of inadvertent arthropod infection and transmission. Whenever feasible, use of sterile blood or blood from sources known to be specific pathogen free is recommended, whereas use of blood from animals or humans whose disease status is uncertain should be avoided. In some instances, a vector colony is specifically adapted to and will not propagate without human blood acquired directly by feeding on a volunteer. Such arthropods should not be fed a second time on a different volunteer; those fed initially by membrane on animal or human blood should not be allowed to subsequently feed on a human volunteer.

### Safety equipment (primary barriers)

#### Gloves

Latex or nitrile gloves should be used when handling host animals or blood used to feed the arthropods, but local risk assessment and institutional policy may provide exceptions.

#### Torso apparel

White laboratory coats, gowns, and/or uniforms should be worn at all times in the insectary when handling blood and vertebrate animals, but local risk assessment and institutional policy may provide exceptions.

#### Arthropod-specific personal protective equipment

Personal protective equipment is worn as appropriate, for example, respirators for arthropod-associated allergies, particle masks, and head covers, but local risk assessment and institutional policy may provide exceptions.

### Facilities (secondary barriers)

#### Location of insectary

The insectary area is separated, if possible, from areas that are used for general traffic within the building.

#### Insectary doors

Door openings, whether covered by rigid panels, glass, screens, plastic sheets, or cloth, minimize escape and entrance of arthropods or pests.

#### Insectary windows

Windows, if present, effectively prevent escape of the smallest arthropods contained within as well as prevent entry of wild arthropods and pests.

#### Lack of an insectary

Arthropods may be maintained at ACL-1 in rooms other than those specifically designed as insectaries. If the facility does not have secondary barriers that would minimize escape or entry of pests, and is not separated from general traffic, specific operating procedures must be developed and tested to mitigate such risks. For example, mosquitoes might be held by a “cage within a larger cage”; removal of adult mosquitoes accomplished by the aspirator manipulated through cage sleeves placed perpendicular to each other and the sample container loaded entirely within the outer cage. Alternatively, entire mosquito containers may be chilled before aspirating individual mosquitoes. Plexiglas glove boxes might also be used for manipulations, particularly if exotic species are maintained. Nonflying species may be manipulated on designated tables or benches in pans within moats of water, and housed in vials or other containers held within a secondary storage container such as a lidded plastic food container.

## Arthropod Containment Level 2

ACL-2 should be practiced if working with exotic and indigenous arthropods infected with BSL-2 agents associated with animal and/or human disease, or that are reasonably suspected of being infected with such agents (diagnostic samples). The PI must perform a risk assessment when deciding whether arthropods are reasonably suspected of being infected with a pathogen. For example, live mosquitoes collected during the course of a disease outbreak and maintained in the laboratory would present more of a risk to laboratory personnel than those that are cold-immobilized or killed before sorting and identifying them for standard surveillance purposes. Uninfected genetically modified arthropod vectors also fall under this level provided the modification has no or only negative effects on viability, survivorship, host range, or vector capacity. ACL-2 builds on the practices, procedures, containment equipment, and facility requirements of ACL-1. It is more stringent in physical containment, disposal, and facility design requirements. Moreover, access is more restricted than ACL-1. The decision to propagate infected exotic arthropods under ACL-2 conditions in active transmission areas or in cases in which establishment is a possibility typically requires that measures that otherwise would only be recommended or preferred must be instituted as policy.

### Standard practices

#### Location of arthropods

Furniture and incubators containing arthropods are located in such a way that accidental contact and release by laboratorians, custodians, and service persons are unlikely. This may be achieved by locating arthropods in dedicated rooms, closets, incubators located out of the traffic flow, or similar measures. Nonflying arthropods such as ticks are typically held in primary containers (vials) that are placed within an environmentally controlled container such as a desiccator or plastic food container; often, this in turn is held within an environmental chamber. Although a dedicated space is recommended for long-term storage of ticks, appropriate risk assessment by the local IBC or other institutional entities, informed by the PI or other experts, may allow for the housing of ticks in noninsectary settings.

#### Supply storage

The area is designed and maintained to enhance detection of escaped arthropods. Equipment and supplies not required for operation of the insectary should not be located in the insectary. All supplies for insect maintenance that must be kept within the insectary are located in a designated area and not on open shelves. It is recommended that a closed storage room, cabinets with tight-fitting doors or drawers, be used. Doors and drawers are opened only for access. Insect diet should be kept in sealed containers.

#### Primary container cleaning and disinfestation

In addition to cleaning cages and culture containers to prevent arthropod escape as in ACL-1, containers are disinfected chemically and/or autoclaved if used for infected material, according to an IBC-approved protocol and/or laboratory standard operating procedures. To reduce the risk of mixing up uncontaminated with potentially contaminated waste by having different methods for disposal, a laboratory may want to consider routine autoclaving, incineration, or other appropriate decontamination of all primary containers.

#### Primary container construction

Cages used to hold arthropods are shatter-proof and screened with mesh of a size to prevent escape. Containers are preferably autoclavable or disposable. Openings designed to prevent escape during removal and introduction of arthropods are recommended.

#### Disposal of arthropods

All life stages of arthropods must be killed before disposal by freezing or other suitable methods. Infected arthropods should be autoclaved, or decontaminated with chemical disinfectants such as 10% bleach or 70% ethanol based on an agent-specific risk assessment. The lack of an autoclave or means of incineration should be evaluated by local risk assessment and appropriate substitutes sought.

#### Isolation of uninfected arthropods

Spread of agents to uninfected arthropods is usually a low risk, given that most infections occur via hematophagy. Containers must be clearly marked to easily distinguish infected from uninfected arthropods. It is good practice to separate infected arthropods in a separate room, if possible, to prevent them from being mistaken as being uninfected.

#### Primary container identification and labeling

As per ACL-1.

#### Prevention of accidental dispersal via sewer or on persons

Before leaving the insectary and after handling cultures and infected arthropods, personnel wash their hands. Care should be taken to not disperse viable life stages into the drainage system. No infected material is disposed through the sewer unless it is decontaminated. Physical barriers (overlapping sheets and screens) or air curtains are recommended as appropriate; personal protective equipment that is reused (laboratory coats, gowns) should be checked for infestation before exiting the insectary.

#### Pest exclusion program

As per ACL-1.

#### Escaped arthropod monitoring

Investigators assess whether escapes are occurring by instituting an effective arthropod trapping program to monitor the escape prevention program. Oviposition traps, ground-level flea traps, oil-filled channels surrounding tick colonies, light traps for mosquitoes, and so on are recommended. Particularly in the case when exotic arthropods are used, exterior monitoring is recommended. Records of exterior captures are maintained. Any evidence of escape should trigger a review of practices and procedures before resuming work.

#### Source and harborage reduction

Harborage and breeding areas are eliminated. Furniture and racks are minimized and can be easily moved to permit cleaning and location of escaped arthropods. Equipment in which water is stored or might accumulate (*e.g*., humidifiers) is screened to prevent arthropod access, or contains chemicals to prevent arthropod survival.

#### Laboratory sharps

Disposable sharps should be discarded in puncture-proof containers or as mandated by institutional policy. Forceps, dissecting probes, and other sharps that are reused should be frequently disinfected by chemical disinfection or flame sterilization.

#### Routine decontamination

Equipment and work surfaces in the insectary are routinely decontaminated with an effective chemical disinfectant.

#### Notification and signage

Persons entering the area should be made aware of the presence of BSL-2 agents in arthropod vectors, but institutions may vary in their policies for security or other reasons. If infected material is present, typically a BSL-2 biohazard sign is posted on the entrance to the insectary, listing all species handled within and is updated whenever new species are introduced or pathogenic infectious agents are present. The hazard warning sign typically identifies the arthropod species, agent(s) known or suspected to be present, lists the name and telephone number of the responsible person(s), and indicates any special requirements for entering the insectary (*e.g*., the need for immunizations or respirators).

#### Procedure design

All procedures are carefully designed and performed to prevent arthropod escape.

#### Safety manual

A site-specific safety manual is prepared, approved by the IBC or other institutional review entities, and adopted. The manual contains emergency procedures, standard operating procedures, waste disposal, and other information necessary to inform personnel of the methods for safe maintenance and operation of the insectary. If the institution does not have formal review committees, the PI or department head should develop laboratory-specific safety manuals.

#### Training

Laboratory personnel are advised of special hazards and are required to follow instructions on practices and procedures contained in the safety manual. Adherence to established safety procedures and policies is made a condition of employment and is part of the annual performance review, if applicable, of every employee. Personnel receive annual updates and additional training as necessary for procedural or policy changes. Records of all training are maintained.

#### Medical surveillance

An appropriate medical surveillance program should be considered, although institutional policy may vary with this requirement. At the minimum, all personnel should be educated by the PI about the risks associated with the specific tasks and experiments, as well as the signs and symptoms of any illness caused by the agent(s) under study. Specialty immunizations or a serum surveillance system may or may not be part of an institutional occupational health program. In general, persons who may be at increased risk of acquiring infection, or for whom infection may be unusually hazardous (*e.g*., immunocompromised), are not allowed in the insectary unless special personal protection procedures are in place to eliminate extra risk.

#### Access restrictions

Routine access is limited to trained persons and accompanied guests. Service persons are made aware of the hazards present and the consequences of arthropod release and contact with agents that may be present.

#### Special arthropod handling containers and areas

Infected arthropods are prevented from release into the laboratory area. A dedicated area for handling infected material is recommended. This is preferably a separate cubicle, walk-in incubator, or screen room. Additional physical barriers (*e.g*., glove box, biosafety cabinet) or procedures (incapacitated arthropods, *e.g*., removing a wing from a mosquito) may be required depending on the local risk assessment. BMBL and other sources (Crampton et al. 1997) may provide specific recommendations that can be adopted or modified according to the local risk assessment.

#### Safe transport in the laboratory

All infectious and potentially infectious samples are collected, labeled, transported, and processed in a manner that contains and prevents transmission of the agent(s). Transfer of arthropods between manipulation and holding areas is in nonbreakable secure containers.

### Special practices

#### IBC and IACUC approval: as for ACL-1

Microbial agents classified at BSL-2 require at the minimum registration with the appropriate institutional entity (Biosafety Office) and many institutions require IBC review and approval before starting any work. Work with recombinant organisms will usually need review and approval. If applicable at the institution, IACUC review may also be required for work with vertebrate hosts. There may be some institutions, for example, in low- or middle-income countries, where these administrative entities are absent or less formally structured, and hence, this guidance may not easily be applied. In such cases, PIs and department leaders benefit from the existence of these guidelines and could institute site-specific policies of their own to ensure the safety of researchers and the surrounding community.

#### Housing of nonarthropod animals

Other animals are not accessible to the arthropods. Animals used as hosts or blood sources generally are not housed with arthropods. If present, they are adequately protected from access by escaped arthropods.

#### Containment during blood feeding

Recommendations for ACL-1 containment of arthropods during blood feeding are more stringently assured by special practices and container design, as recommended by the local risk assessment.

#### Blood source: as per ACL-1

To prevent inadvertent contamination of the clean colony, sources of infection, such as a tube of infected blood, should not be stored in the same refrigerator as a tube of uninfected blood for maintaining uninfected colonies by membrane feeding. Colony arthropods should be maintained in an ACL-1 area and transported to an ACL-2 area for infection; there should be no transport of living arthropods from ACL-2 to ACL-1 without specific local risk assessment.

#### Escaped arthropod handling

Loose arthropods must be killed and disposed, or recaptured and returned to the container from which they escaped. Infected arthropods must not be killed with bare hands and must be manipulated using filtered mechanical or vacuum aspirators or other appropriate means (*e.g*., forceps, paintbrushes, gloved hands).

#### Accidental release reporting

A release procedure is developed and posted. This includes contacts and immediate mitigating actions. Accidents that result in release of infected arthropods from primary containment vessels or that result in overt exposure to infectious material must be reported immediately to the insectary director (PI) who is responsible for ensuring that appropriate and documented action is taken to mitigate the release. The room where the incident occurred is closed off, a warning sign indicating the location, number, and type of material released is prominently posted, and other laboratory personnel are informed until the source is eliminated. Follow-up medical evaluation, surveillance, and treatment are provided as directed by institutional policy and local risk assessment, and written records are maintained.

#### Movement of equipment

All equipment must be appropriately decontaminated and disinfested before transfer between rooms within the insectary, and before removal from the insectary.

### Safety equipment (primary barriers)

Personal protective equipment should be evaluated as part of the local risk assessment. It should be noted that very few infected arthropods are directly infectious by handling; virtually all require exoskeleton disruption or the act of feeding to be hazardous although there are exceptions (body lice excrete feces that contain *Rickettsia prowazekii* or *Bartonella quintana*; newly fed mosquitoes may diurese infectious virus). Clothing (primary as well as safety) should conform to institutional policy, if any, and to the risk assessment by the local IBC. As an example, entering a room containing an environmental chamber holding plastic food containers with tick vials would not require personal protective equipment (PPE), unless the vials were opened and the ticks manipulated. The use of latex or nitrile gloves, although highly recommended, may not be required as a result of risk assessment by the local IBC or equivalent; manipulation of arthropods within a glove box fitted with Hypalon gloves, for example, would not necessarily require additional gloves.

#### Eye and face protection

Appropriate face/eye and respiratory protection is worn by all personnel entering the insectary, if recommended by the local risk assessment.

#### Gloves

Gloves (latex or nitrile) are worn when handling potentially infected arthropods, blood, and associated equipment and when contact with potentially infectious material is unavoidable. Local risk assessment may provide for exceptions, for example, the need for dexterity or tactile control (*e.g*., during the inoculation of suckling mice).

#### Torso apparel

White laboratory coats, gowns, and/or uniforms are typically worn at all times in the insectary when handling vertebrate animals and infected materials. Universal blood precautions (BMBL5) are recommended when blood is manipulated.

#### Personal clothing

Clothing should minimize the area of exposed skin (*e.g*., skirts, shorts, open-toed shoes, sandals, and tee shirts are inadvisable), since this can increase the risk of attracting and being bitten by a loose arthropod.

#### Arthropod-specific personal protective equipment

Other equipment may be required as determined by the local risk assessment. Homogenization of infected arthropods, for example, may require an appropriate respiratory protective device if the procedure is not performed within a biosafety cabinet or glove box.

### Facilities (secondary barriers)

An insectary may simply be a room with a door that may be closed tightly; it may or may not have environmental controls. Dedicated spaces to be used as insectaries are highly recommended, but resources may not exist to permit such arrangements. The use of infected arthropods may be permitted after risk assessment by the local IBC even in the absence of a dedicated space. Ticks, for example, may be safely manipulated within general BSL-2 laboratory settings that are otherwise not considered to be insectaries.

#### Location of insectary

The insectary is separated from areas that are open to unrestricted personnel traffic within the building. It is recommended that this be accomplished by at least two self-closing doors that prevent passage of the arthropods. Increased levels of physical isolation are recommended, for example, separate buildings, wings, and suites. However, the lack of a dedicated insectary should not imply that infected arthropods may not be manipulated; site-specific risk assessments may provide mitigating alternative arrangements. For example, nonflying infected arthropods such as ticks or fleas may be safely manipulated in a dedicated area within a BSL-2 laboratory using a moat system (pan within a pan of water) and accounting for all specimens.

#### Insectary doors

Recommended entrance to the insectary is via a double-door vestibule that prevents flying and crawling arthropod escape. For example, the two contiguous doors must not be opened simultaneously. Internal doors may open outward or be sliding, and are kept closed when arthropods are present. Self-closing doors are highly recommended. Additional barriers (*e.g*., screened partitions, hanging curtains) may be required by the local risk assessment. Alternative arrangements may be specified by local risk assessment in the absence of a dedicated insectary.

#### Insectary windows

Windows are not recommended, but if present cannot be opened and are well sealed. Windows should be resistant to breakage (*e.g*., double paned or wire reinforced).

#### Vacuum systems

If a central vacuum system is installed, each service outlet is fitted with suitable barriers/filters to prevent arthropod escape. Filters are installed to permit decontamination and servicing. Other vacuum devices are appropriately filtered to prevent transfer and exhausting of arthropods.

#### Interior surfaces

The insectary is designed, constructed, and maintained to facilitate cleaning and housekeeping. The interior walls are preferably light colored so that a loose arthropod can be easily located, recaptured, or killed. Gloss finishes, ideally resistant to chemical disinfectants and fumigants, are recommended. Light-colored floors are also highly recommended, smooth and uncovered. Ceilings are as low as possible to simplify detection and capture of flying insects. Inability to conform to these recommendations may be mitigated by other physical or procedural methods as indicated by the local risk assessment. A static glove box with a light-colored interior, for example, may be used to manipulate infected arthropods where the color of walls and floors cannot be easily changed.

#### Floor drains

Floor drains are modified to prevent accidental release of arthropods and agents. If present, traps must be filled with an appropriate chemical treatment to prevent survival of all arthropod stages (*e.g*., mosquito larvae).

#### Plumbing and electrical fixtures

Internal facility appurtenances (*e.g*., light fixtures, pipes, ducting) are minimal since these provide hiding places for loose arthropods. Penetrations of walls, floors, and ceilings are minimal and sealed/caulked. Ideally, light fixtures are flush with the ceiling, sealed, and accessed from above.

#### Heating, ventilation and air conditioning (HVAC)

Ventilation is appropriate for arthropod maintenance, but does not compromise containment of the agent or arthropod. Examples include the following: exhaust air is discharged to the outside without being recirculated to other rooms; appropriate filter/barriers are installed to prevent escape of arthropods; the direction of airflow in the insectary is inward; a progressively negative pressure gradient is maintained as distance from the main entrance increases; fans located in the vestibule and internal corridor can be used to help prevent escape of flying arthropods; and hanging or air curtains are located in vestibules and doorways. Local risk assessments may provide site- and task-specific alternatives to these recommendations, for example, the use of a static glove box in which infected arthropods are manipulated may provide adequate security if directional airflow is not possible.

#### Sterilization equipment

An autoclave is available, conveniently located in rooms containing arthropods within the insectary building. If an autoclave is not available, an appropriate decontamination system or set of practices and procedures may be recommended by the local risk assessment.

#### Sink

The facility has a hand-washing sink with hot water and with suitable plumbing to prevent arthropod escape.

#### Illumination

Illumination is appropriate for arthropod maintenance, and does not compromise arthropod containment, impede vision, or adversely influence the safety of procedures within the insectary. Lighted (or dark) openings that attract escaped arthropods are avoided.

#### Facility compliance monitoring

The facility should be evaluated annually for compliance to ACL-2. The PI or insectary director inspects the facility at least annually to ensure that alterations and maintenance have not compromised the containment characteristics. Adequacy of the practices and facility in view of changes in research protocols, agents, or arthropods is considered.

## Arthropod Containment Level 3

ACL-3 involves practices suitable for work with potential or known vectors that are or are likely to be infected with BSL-3 agents associated with human disease. Arthropods that are infected or potentially infected with BSL-3 pathogens may pose an additional hazard if the insectary is located in an area where the species is indigenous, or if alternative suitable vectors are present, as an escaped arthropod may introduce the pathogen into the local population. ACL-3 builds on the practices, procedures, containment equipment, and facility requirements of ACL-2. It differs in that access is more restricted, and the microbiological containment takes a more prominent role in determining the practices and facilities.

In the United States, the Select Agent Rule (www.selectagents.gov) restricts access to certain pathogens of human or veterinary importance, all classified at BSL-3 or BSL-4. Many of these Select Agents are naturally maintained by arthropods. All possession and use of these restricted agents must comply with the biosecurity requirements promulgated by the United States Title 42 CFR Part 72. Violations are criminal offenses. These revised guidelines do not review the requirements of the Select Agent Rule other than those that may affect practices and procedures needed for safe research with arthropods containing these restricted pathogens. Other countries may have similar restrictions and these should be kept in mind when planning for the development of an ACL-3 facility.

### Comments

An aspect of working with BSL-3 pathogens that needs clarification is the use of biological safety cabinets. The BMBL states that “All procedures involving the manipulation of infectious materials are conducted within biological safety cabinets or other physical containment devices, or by personnel wearing appropriate protective clothing and equipment.” Most workers with BSL-3 agents utilize biosafety cabinets and seem to regard this approach as the standard. Many medical entomologists and vector biologists have therefore been introduced into BSL-3 research under the impression that they must perform all work involving BSL-3 agents within a biosafety cabinet. Manipulating small arthropods in a biosafety cabinet can be extremely difficult. The airflow can blow small arthropods around the cabinet, into the filters, and into inaccessible locations. If working with cold-anesthetized mosquitoes on a chill table, for example, the arthropods can be blown from the table, recover, and then fly around. The airflow may also disrupt the presentation of cues such as body temperature that stimulate host seeking and feeding. The use of a biological cabinet can thus increase the risks associated with working with arthropod vectors. Whereas a cabinet might safely be used to prepare infectious material, the best option may be to perform infectious procedures in a secure area (glove box) within a containment room and not exposed to strong air currents.

Hunt and Tabachnick (1996) recommend that “insects are never manipulated on an open bench.” These workers provide plans to construct a purpose-designed glove box for such work. SALS (The Subcommittee on Arbovirus Laboratory Safety 1980) stated “infection, anesthetization with carbon dioxide, and transfer of arthropods are done in such a manner that risk of infection of workers by aerosols is minimized. This can be accomplished by use of (a) protective clothing and respirators/masks, (b) a biosafety cabinet, or (c) a plastic isolator with sleeve openings with or without an air exhaust.” There may be alternative approaches, such as the use of disposable plastic glove bags; or using an entire room as primary containment with enhanced PPE and multiple physical barriers (*e.g*., nested moats for ticks). Again, the local risk assessment may weigh the costs and benefits of alternatives to the accepted or typical recommendations.

To prevent arthropod escape, arthropod work is performed in a designated area, preferably small and self-contained within the laboratory, for example, a cage-like room constructed of fine mesh (see Arthropod Containment Levels 2, *facilities (secondary barriers)*). In the event of escape, the search area is therefore small, and the chances of locating the escaped arthropod are correspondingly high. When manipulating arthropods that require ACL-3, biosafety cabinets may be inappropriate because of the airflow and reduced humidity. Safe containment of the arthropods is thus achieved through the use of several levels of containment (cages within incubators, and designated insectary areas) within the BSL-3 laboratory, and appropriate procedures (traps, etc.) including those described below. It is recommended that where possible, the researcher take advantage of the safety provided by working within a biological safety cabinet. Procedures such as virus isolation from frozen mosquito pools can be easily performed in a biosafety cabinet (BSL). Glove boxes may also be useful for manipulating small infected arthropods.

Field-collected arthropods from sites of active transmission of BSL-3 agents do not necessarily require manipulation at ACL-3. Such material is considered diagnostic and may be manipulated at BSL-2/ACL-2; identification of specific samples containing viable agents would prompt transfer to BSL-3/ACL-3. Enhanced practices and procedures, however, should be instituted to prevent escape of living samples while they are assayed, or if aerosols may be generated.

Detection of Select Agents within arthropods, animals, and clinical samples requires prompt reporting of the detection to the Select Agent Program and such samples, if viable (or those containing a regulated positive strand RNA virus, which is potentially directly infectious), require secure storage until their disposition as mandated by the Select Agent Program.

### Standard practices

#### Location of arthropods

Furniture and incubators containing arthropods are located in such a way that accidental contact and release by laboratorians, custodians, and service persons do not occur. This is usually achieved by locating arthropods in dedicated BSL-3 rooms, wings, or suites, preferably in incubators, which would serve as an additional layer of containment. Ticks or other less mobile vector arthropods may be held within vials contained in desiccator cabinets or other escape-proof secondary or tertiary housing other than a dedicated incubator. Site-specific risk assessment may provide for other practices.

#### Supply storage

Equipment and supplies not absolutely required for ongoing ACL-3 work are removed from the insectary after appropriate decontamination or are stored in a designated area and not on open shelves. It is recommended that a closed storage room, cabinets with tight-fitting doors or drawers be used. Doors and drawers are open only during access.

#### General arthropod elimination

In addition to measures for general arthropod elimination within the insectary, materials used to wipe or mop are autoclaved before disposal. Only persons trained to work with arthropods and BSL-3 agents and equipped with appropriate PPE clean up spills.

#### Primary container cleaning and disinfestation

Care is taken to disinfest primary containers in a manner that does not create aerosols. All primary containers are autoclaved or incinerated for disposal, or as specified by the site-specific risk assessment.

#### Primary container construction

Cages used to hold arthropods are nonbreakable and screened with mesh of a size to prevent escape. Containers are autoclavable or disposable. Openings are designed to prevent escape during removal and introduction of arthropods. Disposable containers are recommended.

#### Disposal of arthropods

In addition to ACL-2 disposal practices, the outer surfaces of containers are decontaminated before moving the material. All arthropod waste materials are autoclaved or incinerated. Living, infected arthropods should be killed by freezing or other appropriate methods, followed by autoclaving or incineration; those not being used in an experiment should not be allowed to persist alive. An exception would be long-lived or developmentally delayed arthropods such as ticks, which may require extended durations before they may be physiologically capable of feeding again and hence must be kept for weeks or months. The lack of access to an autoclave or incineration device should be handled during local risk assessment to identify the best substitute available.

#### Isolation of uninfected arthropods

Where possible, only arthropods requiring ACL-3 procedures are housed in the ACL-3 insectary. If it is necessary to house ACL-2 or lower arthropods in the ACL-3 insectary, all procedures and practices must meet the ACL-3 standards.

#### Primary container identification and labeling

As per ACL-1.

#### Prevention of accidental dispersal on persons or via sewer

Viable potentially infected arthropod life stages must not leave the laboratory. PPE is doffed as specified by the risk assessment and IBC protocol. Hands are washed on exit; showering out may or may not be required depending on the risk assessment. No material is disposed through the sewer unless appropriately decontaminated, for example, by treatment with 10% hypochlorite or by heat, autoclaving, or incineration.

#### Escaped arthropod monitoring

Ideally, risk assessments identify practices, procedures, and equipment that will prevent escape of any infected arthropod. “Count-in, count-out” procedures have been recommended for ACL-3 and should be implemented if possible. Although this practice can be implemented well for studies of winged adult insects in the absence of a vertebrate host, there are a number of scenarios where such a practice is logistically difficult. For example, ticks, mites, fleas, and lice may be groomed by an infested host and eaten if they are not contained within a feeding capsule. Feeding capsules themselves may partially detach and liberate some of the nonattached arthropods within and thus should not be considered as the only way to safely contain a feeding nonflying arthropod. “Count-in, count-out” also poses difficulty for studies of transovarial transmission. Eggs may be laid but few hatch; larvae may start to develop but then die and be consumed by the remainder. Pupae may develop but adults may not emerge. Finally, motile diminutive arthropods such as mites might be easily miscounted and lead to a false conclusion that an escape had occurred. The risks that might be associated with not using a “count-in count-out” protocol might be balanced by additional barriers to escape, for example, one or more moats containing the cage with the infested animal, as provided by a local risk assessment.

Strong suspicion of an escape by infected arthropods may require facility shutdown and disinfection, or treatment with insecticide. The ACL-3 PI is usually required to promptly notify institutional authorities such as the biosafety officer if an escape is likely. The appropriate response would be the outcome of deliberations by institutional authorities advised by the PI.

Additional measures are taken to measure the effectiveness of the arthropod surveillance program and these are documented. As part of the review and commissioning process of a new facility, the physical integrity and security practices might be tested by a simple release/recapture study. A known number of noninfected arthropods would be released and then these would be recaptured to assess the physical integrity of security barriers. Such an experiment is described by Hunt and Tabachnick (1996). Exterior and within-building monitoring should be considered. Records of exterior captures are maintained.

The Select Agent Rule poses a specific quandary and additional preparations during initial risk assessments as it relates to arthropod escape: the rule requires that all Select Agents be accounted for. Even a simple miscounting may trigger a federal investigation. Arthropods deliberately infected by Select Agents are themselves considered Select Agents and they must be entered into a written inventory and record of disposition must be made for each individual infected arthropod. The failure to account for all infesting infected arthropods (e.g., 1 of 10 infecting ticks is not found when recovering the replete ticks in a moat surrounding a mouse cage) would be considered an environmental release (or theft) and subject to investigation by the Select Agent Program. It might reasonably be concluded that the host may have eaten the infecting arthropod and such a conclusion needs to be recorded in writing and the reasoning or evidence presented. If this contingency is not approved in advance by the institutional oversight committee during the IBC protocol review, the failure to recover all infecting arthropods would be considered a release and an emergency response would likely be triggered, including prompt notification to the Select Agent Program. Hence, ACL-3 work with Select Agents can represent a particularly onerous and difficult scenario for which there is no easy solution.

#### Pest exclusion program

As per ACL-1.

#### Source and harborage reduction

As per ACL-2.

#### Microbiological and medical sharps

As per ACL-2.

#### Routine decontamination

As per ACL-2.

#### Notification and signage

As per ACL-2.

#### Procedure design

All procedures are carefully performed to prevent arthropod escape and the creation of aerosols or splatters. Protocols are practiced with noninfected arthropods/animals and modified before implementation.

#### Safety manual

As per ACL-2.

#### Training

The training required for laboratory personnel under ACL-3 is more detailed and extensive, particularly if the pathogen in use also is a Select Agent. Specific training in containment practices and procedures may be required by institutions and is highly recommended if personnel are not previously experienced. Such training, and annual refresher training, is a requirement of the Select Agent Program and must be documented.

#### Medical surveillance

An institution with an ACL-3 facility should have a medical surveillance or occupational health program. This is a requirement of the Select Agent Rule and is the standard of practice for many U.S. institutions. It is possible, though, that some institutions do not have a formal medical surveillance or occupational health plan and this may apply to programs in resource-poor sites as well. In such cases, the PI should develop a medical response plan and arrange with local health care providers for education of workers or providing care in the event of an exposure. Where there is a formal occupational health program, institutional policies and the specific (usually independent) program dictate the extent of surveillance, whether prophylaxis may be required, and the appropriate response to exposure. Hence, a one-size-fits-all recommendation on surveillance or occupational health is not advisable.

#### Access restrictions

The insectary director limits access to the insectary to the fewest number of persons possible. Personnel who must enter the insectary for program or service purposes when work is in progress are accompanied by trained laboratorians and are advised of the potential hazard to themselves, coworkers, and the potential consequences of arthropod release. Because of the increased risk to nontrained personnel, laboratory staff should perform general cleaning activities that would otherwise be performed by custodial staff. The Select Agent Rule has specific requirements for personnel access to restricted agents, including background checks and suitability assessment. In addition, any laboratory registered to possess and use Select Agents must keep them secure, with at least three physical barriers (locks or other restrictions) in place to deter theft.

#### Special arthropod handling containers and areas

All work is done within a primary barrier. Appropriate biological safety cabinets, other physical containment devices, and/or personal protective equipment are used whenever conducting procedures to infect arthropods with BSL-3 agents, or when handling arthropods. Appropriate designs will consider the life history and behavior of the arthropod and may differ from that required by the agent alone. Such modifications should be made in consultation with biosafety experts. Manipulation of arthropods and, for example, rearing of transovarially infected immature stages are performed in a designated area. SALS suggests “a separate room or double screened area that is separated from the main insectary by rooms having two screened or solid doors that open inward and closing automatically.”

#### Safe transport in the laboratory

As per ACL-2.

### Special practices

#### IACUC and IBC approval: as per ACL-2

Note that the Select Agent Program now requires that all experiments and activities with Select Agents be outlined as part of the registration process and will be reviewed by their administration.

#### Housing of nonarthropod animals

As per ACL-2.

#### Containment during blood feeding

Recommendations for ACL-1 containment of arthropods during blood feeding are strictly assured by special practices and container designs that prevent escape of arthropods.

#### Blood source

As per ACL-1.

#### Escaped arthropod handling

Loose arthropods must be killed and disposed, or recaptured and returned to the container from which they escaped. Infected arthropods are not killed with hands and must be transferred using filtered mechanical, vacuum aspirators, forceps, or other appropriate tools. Only personnel properly trained and equipped to work with designated arthropods and BSL-3 infectious agents are to recover and/or kill escaped arthropods.

#### Accidental release reporting: as per ACL-2

Institutions may have specific reporting requirements. The Select Agent Program must be notified by the institution of any accidental release of a Select Agent.

#### Movement of equipment

As per ACL-2.

#### Inventory of arthropods

In addition to appropriate primary containment cages, when possible, the number of arthropods must be included on the label, and records are maintained to account for all arthropods from the time of transfer to the ACL-3 insectary to the time of termination. The Select Agent Rule has specific inventory requirements for arthropods infected by Select Agents. The naked nucleic acids of positive strand RNA Select Agent viruses are considered by the Select Agent Program to comprise Select Agents and must be stored and documented as required.

### Safety equipment (primary barriers)

BMBL recommends enhanced PPE for BSL-3 studies. Laboratory surfaces and floors, however, should be considered noninfectious because all procedures and manipulations of infectious material must be within a biosafety cabinet or equivalent primary containment device (and hence any contamination of the laboratory should be considered a breach of operating procedures and trigger institutional review). Nonetheless, many institutions and authorities follow a strict policy that the entire BSL-3 facility is to be considered hazardous and maximum PPE must be used for any and all entry. The cornerstone of biosafety is the site- and task-specific risk assessment and ideally this should guide the requirement for PPE. It should be noted that the maximal PPE may impair dexterity that is essential for performing procedures such as the dissection of small arthropods. In such cases, the local risk assessment should carefully weigh the risks presented and the benefits of using the recommended protective gear and identify other means of mitigating risk if the recommendations are modified. If enhanced PPE is required, workers should rehearse procedures working with uninfected arthropods. Indeed, a good safety practice for all BSL-3 work is to practice all procedures within the BSL-3 facility using a BSL-2 substitute agent before undertaking them at BSL-3.

Some ACL-3 facilities may be designed so that the room itself is the primary containment. Special practices and procedures, including specific PPE, would be mandated by the local risk assessment. No recommendations are made herein as to the details of such practices, procedures, or PPE because they would be site and task specific and should be developed as a result of a discussion of risk assessment by institutional officials and the PI. External biosafety consultants and medical entomologists experienced with containment work might be consulted to help develop such recommendations.

#### Eye and face protection

As per ACL-2.

#### Gloves

Personnel wear latex or nitrile gloves when handling infected arthropods or host animals and associated equipment. Gloves are removed aseptically and are changed frequently. Under specific circumstances, and as allowed by institutional review of practices and procedures specific to the site and task, gloves may not be required, for example, in restraining suckling mice for inoculation (tactile cues and dexterity are required for safe execution of this procedure, as well as for humane purposes).

#### Torso apparel

Changing out of street clothes into scrubs, to be worn under PPE, is highly recommended, although such a requirement should be a result of local risk assessment. White laboratory coats, gowns, or jumpsuits should be worn at all times by all personnel entering the insectary. Wraparound or solid-front gowns are typically worn over this clothing. Front-button laboratory coats alone are unsuitable. The gowns are removed and left in the insectary. Before leaving the insectary, scrub suits are removed and appropriately contained and decontaminated before laundering or disposal.

#### Foot apparel

Boot, shoe covers, or other protective footwear and disinfectant foot baths (with appropriate anti-arthropod measures) are available and used where indicated.

Footwear dedicated for use in the ACL-3 facility is highly recommended.

#### Personal clothing

As per ACL-2.

#### Arthropod-specific personal protective equipment

As per ACL-2.

#### Pesticide

Pesticide for emergency use is available in areas in which escape of arthropods is likely.

### Facilities (secondary barriers)

#### Location of insectary

The insectary is strictly separated from areas that are open to unauthorized, untrained personnel within the building by locked doors. These are opened, for example, by key lock, proximity reader, or card key.

#### Insectary doors

Access to the facility is limited to trained, approved personnel by a self-closing and self-locking door. The external insectary entry doors are controlled by a key lock, card key, or proximity reader. Entry into the insectary is via a double-door entry that includes a change room and shower(s). Showers are plumbed to prevent arthropod escape. An additional double-door access (air lock) or double-door autoclave may be provided for movement of supplies and wastes into and out of the facility, respectively. The two contiguous doors must never be opened simultaneously. Internal doors may open outward or be sliding, but are self-closing, and are kept closed when arthropods are present. Additional barriers (*e.g*., hanging curtains) are recommended.

#### Insectary windows

Windows are not recommended. Any windows present are resistant to breakage (*e.g*., double paned or wire reinforced) and well sealed.

#### Vacuum systems

As per ACL-2.

#### Interior surfaces

In addition to the recommendations for ACL-2, spaces around doors are sealed to facilitate decontamination. Troughs surrounding door frames may be installed and filled with sticky or greasy material that will trap crawling arthropods, depending on the species in use and local risk assessment.

#### Floor drains

Floor drains are not recommended. If present, traps must be filled with an appropriate treatment to prevent survival of any arthropod stage (*e.g*., mosquito larvae). Ideally, all drains are plumbed to a holding tank to facilitate heat or chemical treatment to kill all stages of arthropod before disposal into the waste system.

#### Plumbing and electrical fixtures

As per ACL-2.

#### HVAC

Ventilation is appropriate for arthropod maintenance, but does not compromise containment. Exhaust air is discharged to the outside without being re-circulated to other rooms. Exhaust must be dispersed away from occupied areas and air intakes, or the exhaust must be high-efficiency particulate air (HEPA)-filtered. The USDA requires that air supply vents also be HEPA filtered if working with BSL-3 veterinary agents. Appropriate filter/barriers are installed to prevent escape of arthropods. The direction of airflow in the insectary is inward. A progressively negative pressure gradient is maintained as distance from the main entrance increases. Personnel must verify that the direction of the airflow is proper (a visual monitoring device/meter is recommended to confirm directional inward airflow). Audible alarms alert personnel to system failure.

#### Sterilization equipment

An autoclave is available within the suite of rooms containing arthropods. If an autoclave is not available within the suite, local risk assessment may provide for a suitable alternative (heat treatment, tissue digester) or provide protocols for transport of materials to a nearby autoclave.

#### Sink and shower

In addition to the ACL-2 recommendation, an appropriately plumbed shower is available within the insectary suite. If a shower is not available within the suite, local risk assessment may provide for a suitable alternative.

#### Illumination

As per ACL-2.

#### Biosafety cabinets

HEPA-fitted exhaust air from Class II biological safety cabinets can be recirculated into the insectary provided it is certified annually. If Class III cabinets are used they must be installed appropriately and also certified annually. Static glove boxes for arthropod manipulation (Plexiglas or other more expensive materials) do not require negative air pressure, although accommodation for interior air pressure may be needed (*e.g*., 0.22 μm filter vent).

#### Facility compliance monitoring

The completed ACL-3 insectary design and operational procedures must be documented by the PI and reviewed by the IBC. In some circumstances, ACL-3 insectaries are not built *de novo* and must be retrofitted into existing space. The PI should be a member of any design team and the space modified to reflect the likely uses. If certain ACL-3 recommendations are not practical or cannot be implemented, the local risk assessment may provide site- and program-specific mitigation by alternative physical design or added procedural/practice stipulations.

The insectary must be tested for verification that the design and operational parameters have been met before operation. ACL-3 insectaries are reverified by the PI and by the institutional biosafety representatives where applicable at least annually against these procedures. Laboratory design, practices, and procedures may need to be modified iteratively by operational experience.

## Arthropod Containment Level 4

ACL-4 safety guidelines are for the most dangerous pathogen-infected arthropods. All of the Standard Practices of ACL-3 should be in place, with the additional caveats described here. No compromise is acceptable at this level of work. BSL-4 agents are associated with a high risk of infection from aerosol exposure, and cause life-threatening disease. Certain other pathogens such as those listed as “restricted animal pathogens” may also necessitate BSL-4 containment if used in vectors. For vector work, production of aerosols is a potential risk when preparing infectious meals or inocula, and can also result from analytical practices involved in virus isolation. If work with vectors must be performed in a BSL-4 facility, then BSL-4 requirements must be strictly followed. As described below, vectors must be safely contained at all times possibly by use of specially designed apparatus that is tested and approved before use.

Although arthropods collected from sites where BSL-4 agents are known to occur should be handled with extreme care, they are by definition diagnostic samples and can be handled at ACL-2 in field laboratories. It would be excessive to suggest, for example, that malaria entomology programs within Ebola virus endemic sites be required to handle field-collected parous *Anopheles* spp. within ACL-4; of course, appropriate caution should be taken to prevent exposure to blood regardless of source (universal precautions). Any identification of a BSL-4 agent within such samples requires that the sample be transferred to an ACL-4 facility or treated so that the agent is no longer viable. It should be noted that RNA from a Select Agent that is a positive sense RNA virus is considered to be a Select Agent because of a theoretical consideration of infectivity (by transfection into cells). Because BSL-4 agents are exotic, importation of field-derived materials into the United States will require import permits from CDC and USDA/APHIS, which will have specific stipulations, perhaps including the need for unpacking and manipulating imported materials only within ACL-4 regardless of diagnostic status.

Of the 12 infections requiring BSL-4 containment in the United States, 5 are transmitted by arthropods: Congo/Crimean hemorrhagic fever, Kyasanur Forest disease, Omsk hemorrhagic fever, Far Eastern tick-borne encephalitis, and Siberian tick-borne encephalitis. All of these viruses are Select Agents. Only ticks have been implicated in their natural transmission cycles, although other arthropods have been experimentally infected with BSL-4 agents (*e.g*., *Aedes aegypti* with Marburg, and mesostigmatid mites with Junin). With this information one might only consider measures and protocols that safely contain species of ticks as relevant to BSL-4 research with arthropods. However, new infections may emerge, and thus, it is necessary to consider other arthropods that might require high containment, particularly flying insects. Furthermore, research on newly discovered pathogens often requires experimental attempts to infect arthropods in an attempt to determine the life cycle.

As the number of BSL-4 laboratories is quite limited, the reader should refer to the appropriate sections of the BMBL. For arthropod work, a simple, minimalist approach is adopted. An area designated for arthropod research is small, light colored, and contains only items required for the study. There are two types of BSL-4 laboratories: (1) the cabinet laboratory where the agent is handled in a Class III biological safety cabinet and (2) the suit laboratory. Personnel working in a BSL-4 suit facility don one-piece positive pressure personnel suits ventilated by a life support system. Construction of a BSL-4 facility, and required operating procedures, is sufficient to guarantee that no life stage could survive, and escape from primary containment is mitigated by efficient secondary containment.

Ideally, under ACL-4, an infected arthropod must never be handled outside of a primary containment barrier; for example, cages are opened only in an arthropod-secure glove box (Hunt and Tabachnick 1996). Although a glove box is highly recommended for all manipulations of live arthropods at ACL-4, wearing a positive pressure containment suit would make such a recommendation difficult in practice. Well-lit, stand-alone handling tables incorporating a moat to prevent escape could be used in the open BSL-4 laboratory for manipulating ticks or mites. Flying insects would pose a containment difficulty and the specific experiment may need to custom practices, procedures, and equipment to safely carry out the required tasks.

At ACL-4, every arthropod is counted and accounted for throughout the experiment. No one enters or leaves the room until all arthropods are accounted for and, if living, secured in double-taped cages or vials and placed in secondary sealed holding trays. Infected arthropods will be discarded (killed and decontaminated), processed for analysis (preferably killed and held under conditions that inactivate the agent), or held alive in primary and secondary secure containers. If one arthropod is missing and cannot be found, the facility is shut down and treated with a pesticide.

The nature of this research and the protective equipment required dictate that staff must be trained to the very highest level. Since working with arthropods often requires the use of small instruments and hence considerable dexterity, it is recommended that a specific person be designated for this work and be trained extensively using a space suit so that they are well rehearsed before actual ACL-4 work. Equipment that is used for ACL-3 work will be specially adapted for ACL-4 research, and such work would require extensive practice.

## Transportation and Transfer of Biological Agents and Arthropod Vectors

Transportation refers to the packaging and shipping of materials by air, land, or sea, generally by a commercial conveyance. Transfer refers to the formal process of exchanging these materials between facilities.

Biological agents include infectious agents of humans, plants, and animals, as well as the toxins that may be produced by microbes and by genetic materials potentially hazardous by themselves or when introduced into a suitable gene delivery agent. Etiologic agents and infectious substances are closely related terms that are found in the transfer and transportation regulations. Biological agents may exist as purified and concentrated cultures and may also be present in a variety of materials such as body fluids, tissues, and soil samples. Arthropod vectors are organisms such as mosquitoes, ticks, and fleas that may transmit infectious agents to animals or humans. Biological agents and materials and vectors that are known or suspected to contain them are recognized by federal and state governments as hazardous materials, and their transportation and transfer are subject to regulatory control. Transport and transfer of live, uninfected vectors may also be subject to federal and state regulatory control.

### Transportation

Regulations on the transportation of biological agents and live vectors are aimed at ensuring that the public and the workers in the transportation chain are protected from exposure to any agent that might be in the package, and that the package prevent escape of the agent or live vector. Protection is achieved through (1) the requirements for rigorous packaging that will withstand rough handling and contain all liquid material within the package without leakage to the outside; (2) appropriate labeling of the package with the biohazard symbol and other labels to alert the workers in the transportation chain to the hazardous contents of the package; (3) documentation of the hazardous contents of the package should such information be necessary in an emergency situation; and (4) training of workers in the transportation chain to be able to respond appropriately to emergency situations. Regardless, nonmotile forms such as eggs or nonflying stages should be shipped if possible.

### Transportation Regulations

#### Public Health Service 42 CFR Part 72: Interstate Transportation of Etiologic Agents

Harmonizes with the other U.S. and international regulations [see Federal Register 64(208) p. 58022 at www.access.gpo.gov]. A copy of the current regulation can be obtained from The Code of Federal Regulations (INTERSTATE SHIPMENT OF ETIOLOGIC AGENTS 1, 1980).

#### Department of Transportation: 49 CFR Parts 171–178—Hazardous Materials Regulations

Applies to the shipment of both biological agents and clinical specimens. Information may be obtained from the Internet: www.gpo.gov/fdsys/pkg/CFR-2012-title49-vol2/xml/CFR-2012-title49-vol2-subtitleB-chapI-subchapC.xml (accessed 12/8/2017).

#### United States Postal Service: 39 CFR Part 111—Mailability of Etiologic Agents

Codified in the Domestic Mail Manual 124.38: Etiologic Agents Preparations (www.gpo.gov/fdsys/pkg/FR-2003-06-06/html/03-14185.htm accessed 12/8/2017).

#### Occupational exposure to blood-borne pathogens: Occupational Health and Safety Administration (OSHA)—29 CFR Part 1910.1030

Provides minimal packaging and labeling requirements for transport of blood and body fluids within the laboratory and outside of it (www.osha.gov/pls/oshaweb/owadisp.show_document?p_table=STANDARDS&p_id=10051 accessed 12/8/2017).

#### Dangerous Goods Regulations: International Air Transport Association

These regulations provide packaging and labeling requirements for infectious substances, materials, clinical specimens that have a low probability of containing an infectious substance, and live vectors. These are the regulations followed by the airlines and are therefore of particular relevance for express shipment of arthropods. These regulations are derived from the Committee of Experts on the Transport of Dangerous Goods, United Nations Secretariat, and the Technical Instructions for the Transport of Dangerous Goods by air provided by the International Civil Aviation Organization (ICAO). A copy of the Dangerous Goods Regulations (DGR) may be obtained by calling 1-800-716-6326 or through the Internet: www.iata.org/publications/pages/index.aspx (accessed 12/8/2017).

### General packaging requirements for transport of live arthropod vectors

Transport of live arthropod vectors requires packaging that prevents the escape of arthropods and agents, maintains their viability, and protects personnel in the transportation chain from exposure to the contents. This is true regardless of whether or not the arthropods are infected. Fortunately, unlike many larger animals, most arthropod vectors do not require large containers, ventilation, feeding, or added water during their transport. Most are shipped without free water so the possibility of leaking is rare, and the container temperatures normally maintained during shipments are adequate.

This means that appropriate physical packaging of vector arthropods is fairly simple and, for infected arthropods, can be similar to that which is appropriate for the agents they contain. The following section is intended to provide specific instructions for determining the type of container and labeling required for shipment of vector arthropods.

International Air Transport Association (IATA) Live Animal Regulations (LAR) 42nd edition (available for purchase at www.iata.org/publications/pages/index.aspx, accessed 12/8/2017) describes containers that are appropriate for the shipment of arthropods, including insects and arachnids. The design of these, while not as demanding, is consistent with containers used to ship etiologic agents (see Container Requirement 62 of LARs). It is therefore possible to select containers that satisfy the requirements of LARs, DOT 49 CFR Part 173.196: Transportation of Etiologic Agents, and USPHS 42 CFR Part 72—Interstate Shipment of Etiologic Agents.

According to the IATA DGR, a live, intentionally infected animal that is known to contain an infectious substance cannot be transported by air unless it cannot be transported by any other means. A specific exemption from DOT must be obtained. It should be noted that although infected vectors pose different kinds of risks than do infected vertebrates, any hazard does not exceed that of the infectious agents that are allowed for transport under the DGR. Consulting with transportation authorities or transport companies is strongly suggested in advance of shipping of any infected arthropods.

Three packaging scenarios are considered:

- Arthropods free of infection by specific pathogens.
- Domestic and exotic arthropods containing a nonselect agent.
- Domestic and exotic arthropods containing a select agent.

#### Definitions

##### Domestic arthropods

Those that are extant in the 49 continental United States. Note that this differs from the definition used in Risk Assessment and Containment Levels.

##### Exotic arthropods

All others.

##### Select agent

Etiological agents listed in 42 CFR Part 72.

##### Nonselect agent

Agents other than those above that are known to cause disease in humans.

- Noninfected exotic and domestic arthropods that vector disease are packaged consistently with the minimum packaging requirements of 42 CFR 72.2. This requires that the container must prevent “leakage (*i.e*., escape, note added) of the contents, shocks, pressure changes, and other conditions incident to ordinary handling in transportation.” We recommend that this consists three levels of containment, including a primary receptacle consisting of a sealed plastic bag or tube surrounded by padding, a secondary container such as an insulated chest whose lid is sealed with tape, and a durable fiberboard, wood, plastic, or wooden outer container. The container may bear the “live animal” label naming the species within. If aquatic stages are shipped, the container should also contain sufficient absorptive material to absorb and contain all of the water.
- Domestic and exotic arthropods containing a nonselect agent are packaged as above. The outer container bears a “biohazard” label as described in CFR 72.3. An itemized description of the contents is placed between the outer and inner containers.
- Domestic and exotic arthropods containing a select agent are packaged, labeled, and tracked as required for the agent they are known or suspected to contain. This includes all attendant regulations required for the agent alone, including notice of delivery and failure to receive, and laboratory registration.

### Transfer

Regulations on the transfer of biological agents and live vectors are aimed at ensuring that the change in possession of biological materials is within the best interests of the public and nation. These regulations require documentation of the personnel, facilities, and justification of need for the biological agent in the transfer process, and subsequent approval of the transfer process by a federal authority. The following regulations fit in this category.

## Importation of Etiologic Agents of Human Disease and Live Vectors

### 42 CFR Part 71 Foreign Quarantine

#### Part 71.54 etiologic agents, hosts, and vectors

This regulation requires an import permit from the CDC for importing etiologic agents of human disease, any materials that may contain etiologic agents, including live animals and live vectors. This regulation also requires that an import permit be obtained by the recipient for transfer from the original permit holder of an imported etiologic agent or live vector within the United States. An application and information on importation permits may be obtained at: www.cdc.gov/od/eaipp/importapplication/agents.htm (accessed 12/8/2017).

Interstate transfer of biological agents and live vectors may also be restricted by state regulations. Shippers and recipients of these materials may obtain additional information directly from state health or agriculture departments.

## Importation of Etiologic Agents of Livestock, Poultry, and Other Animal Diseases

### 9 CFR Parts 92, 94, 95 96, 122, and 130

These regulations require an import permit from the USDA, APHIS, and Veterinary Services to import or domestically transfer etiologic agents of livestock, poultry, other animals, and any materials that might contain these etiologic agents. Information may be obtained at (301) 734-3277, or from the Internet: www.aphis.usda.gov/aphis/ourfocus/animalhealth/animal-and-animal-product-import-information/ct_organisms_and_vectors (accessed 12/8/2017).

## Transfer of Select Biological Agents of Human Disease

### 42 CFR Part 72.6 additional requirements for facilities transferring or receiving select agents

Facilities transferring or receiving Select Agents must be registered with the CDC and each and every transfer of a Select Agent must be approved by the Select Agent Program. Information may be obtained on the Internet: www.selectagents.gov (accessed 12/8/2017).

## Export of Etiologic Agents of Humans, Animals, Plants, and Related Materials

### Department of Commerce: 15 CFR Parts 730–799

This regulation requires that exporters of a wide variety of etiologic agents of human, plant, and animal diseases, including genetic material, live vectors, and products that might be used for culture of large amounts of agents, must obtain an export license. Information can be obtained by calling the Department of Commerce (DoC) Bureau of Export Administration at 202-482-4811 or through the Internet: www.bis.doc.gov/index.php/regulations/export-administration-regulations-ear (accessed 12/8/2017).

## Appendix 1. Draft Committee Members v.3.1

*Kate Aultman, NIH

Abdu Azad, U MD

*Ben Beard, CDC, Atlanta, GA

*Mark Benedict, CDC, Atlanta, GA

John Beier, Tulane U.

Al Handler, USDA-ARS, Gainesville, FL

*Steve Higgs, UTMB, Galveston, TX

Peter Jahrling, USAMRID

Anthony James, UC Irvine

Cynthia Lord, FMEL

Robert McKinney, NIH

*Roger Nasci, CDC, Ft. Collins, CO

Ken Olson, CSU, Ft. Collins, CO

Jonathan Richmond, CDC, Atlanta, GA

*Tom Scott, UC Davis

*Dave Severson, U Notre Dame

Walter Tabachnick, FMEL, Vero Beach, FL

Ned Walker, Mich. St.

*Dawn Wesson, Tulane U.

* Lead drafting authors

## Appendix 2. Drafting Process v.3.1

A subcommittee appointed by the American Society of Tropical Medicine and Hygiene (ASTMH)/American Committee of Medical Entomology (ACME) at the 1999 meeting in Washington, DC (above), consisted of persons selected or who volunteered to serve on the committee. They were charged with the task of formulating Draft Guidelines reflecting the containment principles of documents that are currently circulating both in the United States and internationally. The document was intended to address the following items: scope and intent, principles of risk assessment, definition of risk levels considering diseases vectored, phenotype and genotype including that of transgenics, biological containment, risk relative to that in existence due to accidental escape, and containment facilities, practices, and shipping methods appropriate to each risk level.

During the spring of 2000, the Draft Committee formulated a first draft and circulated it among the membership of the committee for comments and revision. After comments were received and considered, a second draft was written (v 2.1). This draft was circulated electronically in numerous places, including the Vector, Mosquito-L, and Biosafety listservers, and was also posted on ProMED. Additional copies were distributed electronically to individuals identified by the Draft Committee as being influential and knowledgeable in the area. Several persons were identified to review and comment on the Guidelines to the Draft Committee at the 2000 ASTMH meeting in Houston, TX.

That meeting was held as an open meeting both to present the guidelines and to receive comments.

National Institutes of Health (NIH) and Centers for Disease Control and Prevention (CDC) Office of Health and Safety personnel and other biosafety experts examined the document and revised it before ACME approval. The guidelines were published in June 2003 as a special issue of Vector Borne Zoonotic Diseases, volume 3, number 2.

## Appendix 3. Description of Revisions

### Draft 3.2

#### October 15, 2015

Noting that it has been nearly 15 years since the last revision, at the 2013 American Committee of Medical Entomology (ACME) Council meeting, a subcommittee was formed to examine and update the guidelines. The subcommittee comprised Sam Telford (Tufts University), chair; Michael Turell (USAMRIID); Kevin Macaluso (Louisiana State University); and Philip Armstrong (Connecticut Agricultural Experiment Station). The entire document was examined and the actual recommendations edited and modified to reflect the experience accumulated with operating under version 3.1. Additional edits were made by Lyric Bartholomay (UW-Madison) and Philip Armstrong (Connecticut Agricultural Experiment Station) and were reviewed and approved by the ACME taskforce on advocacy. The ACME Executive Council unanimously endorsed its publication in 2018.

#### Changes since Draft 3.1

Minor editorial and grammatical changes were made throughout the text. The major additions and deletions include the following:

(1) The major effects of the Select Agent Rule on work with arthropods were specifically incorporated.
(2) Recommendations were modified or added to better address nonflying arthropod vectors (ticks, fleas, mites, fleas), a gap in the prior versions.
(3) Language has been added and grammar modified to reemphasize that the arthropod containment level (ACL) guidelines are recommendations and not regulations and that site- and task-specific modifications are based on a local risk assessment.
(4) Language has been added to address diagnostic samples. The recent publication of guidance for clinical diagnostic laboratories is referenced because it contains excellent discussions of risk assessment and mitigation.

### Draft 3.1

#### December 20, 2001: changes since Draft 3.0

Removed ACL-F designation and text. Moved field site information to the Intent section. Removed section Importation of Plant Pests. Minor changes in grammar and wording. Removed citation to Sullivan, Songer and Estrem. Submitted to the ACME Executive Council for consideration regarding publication. Submitted to the Centers for Disease Control and Prevention (CDC) and National Institutes of Health (NIH) Offices of Health and Safety for consideration and comment. Resubmitted to Mary Bartlett (CDC/DPD editor) for detailed editing.

### Draft 3.0

#### February 15, 2001: changes since Draft 2.3

Added ACL-F designation and text. Added language in risk assessment regarding autonomous transposable elements in transgenic arthropod experiments. Minor grammatical errors corrected.

### Draft 2.3

#### November 14, 2000: changes since Draft 2.2

Added URLs, minor wording changes. (See e-mail to committee of November 14, 2000, for details.)
